# Effects of Heatwaves on Hospital Admissions for Cardiovascular and Respiratory Diseases, in Southern Vietnam, 2010–2018: Time Series Analysis

**DOI:** 10.3390/ijerph20053908

**Published:** 2023-02-22

**Authors:** Nguyen Thi Trang Nhung, Le Tu Hoang, Tran Thi Tuyet Hanh, Luu Quoc Toan, Nguyen Duc Thanh, Nguyen Xuan Truong, Nguyen Anh Son, Hoong Van Nhat, Nguyen Huu Quyen, Ha Van Nhu

**Affiliations:** 1 Faculty of Fundamental Sciences, Hanoi University of Public Health, Hanoi 10000, Vietnam; 2 Faculty of Environmental and Occupational Health, Hanoi University of Public Health, Hanoi 10000, Vietnam; 3 Ministry of Health, Hanoi 10000, Vietnam; 4 Institute of Hydrology and Meteorology Science and Climate Change, Hanoi 10000, Vietnam; 5 Faculty of Basic Medicine, Hanoi University of Public Health, Hanoi 10000, Vietnam

**Keywords:** heatwaves, hospital admissions, cardiovascular, respiratory, time-series analysis

## Abstract

This study investigated the associations between heatwaves and daily hospital admissions for cardiovascular and respiratory diseases in two provinces in Viet Nam known to be vulnerable to droughts during 2010–2018. This study applied a time series analysis with data extracted from the electronic database of provincial hospitals and meteorological stations from the corresponding province. To eliminate over-dispersion, this time series analysis used Quasi-Poisson regression. The models were controlled for the day of the week, holiday, time trend, and relative humidity. Heatwaves were defined as the maximum temperature exceeding P90th over the period from 2010 to 2018 during at least three consecutive days. Data from 31,191 hospital admissions for respiratory diseases and 29,056 hospitalizations for cardiovascular diseases were investigated in the two provinces. Associations between hospital admissions for respiratory diseases and heatwaves in Ninh Thuan were observed at lag 2, with excess risk (ER = 8.31%, 95% confidence interval: 0.64–16.55%). However, heatwaves were negatively associated with cardiovascular diseases in Ca Mau, which was determined amongst the elderly (age above 60), ER = −7.28%, 95%CI: −13.97–−0.08%. Heatwaves can be a risk factor for hospital admission due to respiratory diseases in Vietnam. Further studies need to be conducted to assert the link between heat waves and cardiovascular diseases.

## 1. Introduction

Cardiovascular disease (CVD) was ranked as the greatest single contributor to the overall disease burden in the world. In 2017, CVDs were responsible for 17.79 million deaths globally and 201.14 thousand deaths in Vietnam [1]. Respiratory disease (RD), such as pneumonia and acute lower respiratory diseases, was the main cause of death among the elderly [1]. Extreme ambient temperature is an important environmental hazard and associated with an overall mortality of respiratory diseases and CVDs [2,3].

Heatwave is defined as a period when the weather is hotter than usual [4,5], and a significant public health hazard [4,5,6,7]. Heatwaves might affect many human organ systems such as neurological and physiological systems, then lead to hospital admissions, and even death in vulnerable groups. For mortality, Cheng, et al. [8] indicated that heatwave is associated with a 14.9% increase in cardiovascular and 18.3% respiratory mortality in China, 11% cases of mortality for all-natural causes in Korea [9], and 2% in Australia [10]. In addition, heatwave‘s effect on morbidity includes hospital admissions for many reasons, including stroke or chronic obstructive pulmonary diseases in adults and respiratory diseases in children. While Hopp depicted relevant diagnoses of heatwave-related hospital admissions in the United States, especially cardiovascular diseases [11], Xu et al. [12] reported that heatwave intensity increased significantly the hospital admissions of infants in Brisbane, Australia. However, not many studies have been conducted in lower- and middle-income countries that have tropical climates, owing to poor data systems and lack of research funds on this topic.

The Global Climate Risk Index 2020 ranked Vietnam, a tropical developing country in Southeast Asia, as the sixth country in the world most affected by climate variability and extreme weather events over the period 1999–2018 [13]. Over the past four decades, there were noticeable changes in Vietnam’s climate, particularly increasing temperatures [14]. The mean annual temperature has increased by 0.62 °C since 1958, with the rate of increase more rapid in the dry seasons and more intense in the southern parts of the country [14]. Extreme temperature and the number of ‘hot’ days are projected to have an upward trend, with the largest increases likely in the North Central Coast, South Central Coast (including Ninh Thuan Province), and Southern Vietnam (including Ca Mau Province). Droughts could become more severe due to rising temperatures and rainfall deficits in the dry season [14].

Previous studies evaluated the relationship between heatwaves and hospitalizations in multiple provinces across Vietnam, except Ninh Thuan. The findings in Ca Mau indicated negative associations between heatwaves and admissions for respiratory diseases and CVDs in this province. Ninh Thuan and Ca Mau provinces experienced severe droughts during 2015–2016 and the Vietnam authorities announced an emergency in the region. As such, we conducted a time-series study to investigate the association between heatwaves and hospitalization due to respiratory diseases and CVDs in two provinces, Ninh Thuan in Central Vietnam (a province that frequently experiences heatwaves) and Ca Mau in Southern Vietnam. The results of this study provide insight into developing appropriate responses to heatwaves in these two provinces as well as in similar settings in other countries to help reduce the burden of RDs and CVDs. The study received approval from the Ethical Review Board of Hanoi University of Public Health (Decision No. 371/2018/YTCC-HD38).

## 2. Materials and Methods

### 2.1. Study Area and Population

Ninh Thuan is a coastal area of South-Central Vietnam with a total area of 3355 km^2^ and 607,000 inhabitants in 2017 [15]. Compared with other provinces in Central Vietnam, the weather in Ninh Thuan province is moderately dry with a monthly average temperature ranging from 27.2 °C to 30.2 °C and a monthly average rainfall of 199 mm. Figure S1 of the supplemental document presents maps of these two provinces. Ca Mau, located in Southern Vietnam, belongs to the Mekong Delta region and the southernmost part of Vietnam. The population of Ca Mau was approximately 1,226,300 inhabitants in 2017, living in 5221.2 km^2^. Ca Mau has a subtropical monsoon climate with a short dry season from January to March. The average temperature in Ca Mau was about 27.2 °C from 1979 to 2017. The highest temperature in Ca Mau was recorded at 38.3 °C in 1983.

### 2.2. Data Source

Daily hospital admissions were obtained from the electronic database of two provincial hospitals from 1 January 2010 to 31 December 2017 in Ninh Thuan and 31 May 2013 to 31 July 2018 in Ca Mau (one hospital per province). The provided data period depended on what was available in the available electronic database. Individual data collected included residential locations of the patients, age (or year of birth), gender, primary and discharge diagnoses (coded in International Classification of Diseases version 10-ICD10), and admission and discharge dates. While the location or address of the patients in the electronic database were self-reported information, the diagnosis and the ICD code were provided by the physicians. In this analysis, we only examined data from patients living in Ninh Thuan or Ca Mau when they were hospitalized.

We calculated the daily counts of hospital admissions for two outcomes: CVDs and RDs. In this analysis, patients with ICD codes J00–J99, excluding J60–J70 (lung diseases due to external agents and J92 (pleural plague), were coded as hospital admissions for respiratory diseases. Patients with ICD10 codes from I00 to I99, excluding I00 to I02 (acute rheumatic fever) and J05 to I09 (chronic rheumatic heart diseases), were coded as hospital admissions for CVDs. The codes used in this definition were primary codes at admission. Since daily means of hospital admissions for CVDs in Ninh Thuan were quite low (fewer than one case per day), the analysis for CVD in Ninh Thuan was not conducted.

The meteorological data were provided by the corresponding provincial hydrometeorological stations, Phan Rang and Ca Mau. Daily data variables include the mean, minimum, maximum temperature (Celsius degree—°C), the daily mean relative humidity (%), the daily amount of sunshine (hour), and rainfall level (mm). Due to various existing heatwave definitions [16], we defined heatwave in this analysis as periods of at least three consecutive days when the maximum temperature exceeded the 90th percentile. This definition has been used by previous studies in Vietnam [17] and China [18,19].

### 2.3. Data Analysis

Generalized additive Quasi-Poisson log-linear models were used to assess the increased risk of hospitalization during heatwave days compared to non-heatwave days, controlling for long-term trends and seasonality. The associations in each province were investigated separately. The core model—a model with only a time trend—was built at first. We used Partial autocorrelation function plots (PACF) of the residuals to determine the degrees of freedom of the spline function to minimize the residual serial correlation [20]. We used a flexible spline function with five and four degrees of freedom per year to adjust for long-term trends and seasonal variations in hospitalizations in Ninh Thuan and Ca Mau, respectively. Moreover, models were adjusted for the natural cubic spline function of relative humidity with three degrees of freedom, categorical variables for day of the week, holidays, and daily mean rainfall. The other potential meteorological factors were added based on Generalized Cross-Validation (GCV) values. Holidays were defined as government holidays and were added to the models since the capacity of the hospital during the holiday periods might differ from normal workdays. Heatwaves were added to the final models.

To investigate the delayed acute effects, the associations between heatwaves and hospital admissions for lag 0–lag 4 were reported. Sensitivity analyses were conducted with a variety of degrees of freedom for the long-term trend, df = 3, 4, 5, 6, 7, and restricted the data to warm seasons (from April to September). The risks were presented in the percentage change (excess risk—ER % = (Risk Ratio-1) × 100%) in the number of hospitalizations for respiratory diseases or CVDs two days after the heatwave occurred. Statistical significance was defined as a two-tailed *p*-value smaller than 0.05. All statistical analyses were conducted using R (version 1.2.5019, http://www.r-project.org, accessed on 3 December 2020), using the “mgcv”, “spline” and “gam” packages.

## 3. Results

Table 1 presents descriptive statistics of the admissions. In total, 31,191 hospital admissions for respiratory diseases and 29,056 hospitalizations for CVDs were investigated in two provinces between 2010 and 2018. The average daily admission for RDs in Ninh Thuan and Ca Mau was 6.2 and 7.0 cases, respectively. On the other hand, the average daily count of admission for CVDs in Ca Mau was 15.4 cases. The elderly (age 60+) had more admissions than the younger group (ages 6–60).

The number of admissions during heatwaves in two provinces is presented in Table 1. While in Ninh Thuan, the number of admissions during heatwaves was likely higher than on normal days, the number of admissions during heatwave days in Ca Mau was slightly lower for the entire study period. For instance, daily admissions for respiratory diseases during heatwaves in Ninh Thuan were 6.3 cases per day for ages 6–60 and 2.6 cases per day for those aged 60+. In Ca Mau, there were 12.7 admissions per day for CVDs when a heatwave occurred.

Table 2 presents a summary of meteorological factors and heatwaves in Ninh Thuan and Ca Mau. In Ninh Thuan, 34 heatwaves occurred from 2010 to 2018 with a mean duration of about 6.4 days per time. The maximum duration of the heatwave was 17 days, recently occurring in 2016 and 2017. Furthermore, during a heatwave, relative humidity and rainfall levels were lower than the entire period while the hours of sunshine were longer.

In Ca Mau, on the other hand, there were 18 heatwaves between 2013 and 2018. However, the duration of each heatwave was longer than Ninh Thuan, with a mean of 7.8 days. Indeed, a heatwave occurring in 2015 extended for a month (18 April to 22 May), and another in 2016 extended for more than a month (9 April to 15 May). As with Ninh Thuan, relative humidity and rain level during heatwave periods were significantly lower than the entire study period.

Hospital admissions for respiratory diseases in Ninh Thuan were associated with heatwaves at lag 2, ER = 8.31% (95% CI: 0.64–16.55) (Table 3). However, we did not observe the association between heatwave and hospitalizations for respiratory diseases in Ca Mau. The study observed a negative association between hospitalization for CVDs in Ca Mau. Though the findings did not suggest significant associations in the elderly, the effects amongst them were likely stronger than that in ages 6–60 in two provinces. Indeed, we found a significant link between heatwaves and hospitalization for CVDs in adults aged above 60 in Ca Mau, ER = −7.28, (95% CI: −13.97–−0.08), but not for younger ages.

**Figure 1:**
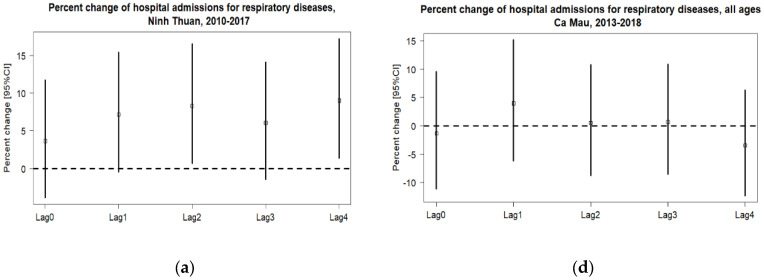

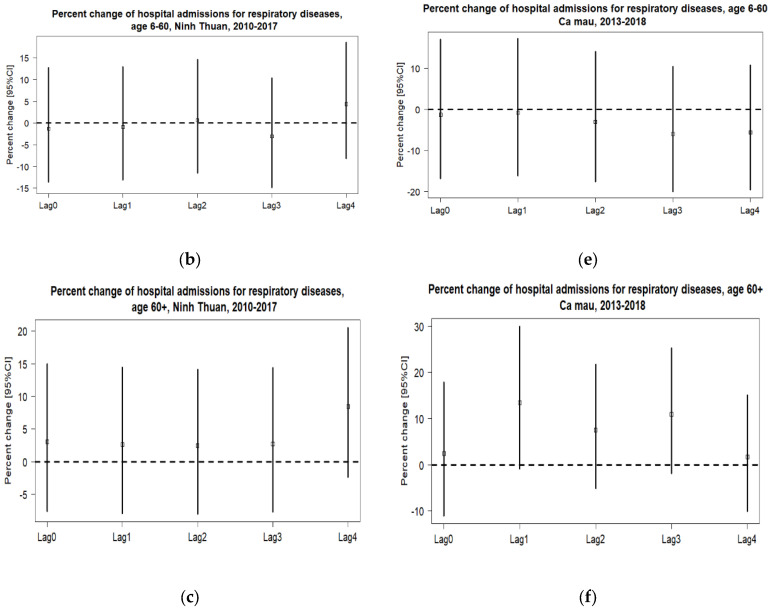
The lag distribution of the associations between heatwave and hospital admissions for respiratory diseases in Ninh Thuan ((**a**) among those aged 6-60; (**b**) among those 60 and above) and Ca Mau ((**c**) among thosed aged 6-60; (**d**) among those 60 and above). Note: Percent changes in the odds of daily hospital admissions were estimated from Quasi-Poisson regression models, adjusting for long-term trends and seasonal variation, day of the week, holiday, and mean humidity (natural cubic spline with four degrees of freedom). While hospital data in Ninh Thuan are from 1 January 2010 to 31 December 2017, hospital data in Ca Mau are from 31 May 2013 to 31 July 2018. Bar: 95% confidence interval.

Figure 1 shows the association between heatwave and hospital admission for respiratory diseases in Ninh Thuan and Ca Mau by lags. In Ninh Thuan, the strongest effect was at lag 4. On the other hand, the study observed the strongest effects on hospitalization for respiratory diseases at lag 1 in all studied ages and two age groups. However, the pattern for CVDs differed. The negative associations between ages 6–60 and 60+ were lost at lag 3 and lag 4 (Figure S2 in the Supplemental Material). The effects on CVDs in age 6–60 was observed at lag 2 and the elderly at lag 4.

Figure S3 in the Supplemental Material presents the association of varying degrees of freedom for time. While the models were consistent for Ninh Thuan, the models were robust for Ca Mau in both outcomes. The effects declined as the degrees of freedom decreased to five or six in models for respiratory diseases and both 6–60 and 60+ age groups (Figure S4 in the Supplemental Material). Indeed, the effects convert from positive association to negative association in models for respiratory diseases and ages 6–60. Nonetheless, the strongest effect was observed at 7 degrees of freedom. Similarly, the effects increased as degrees of freedom increased in the models for CVDs.

Figure S5 compared the estimated effects from the entire data set and restricted data. The restricted data included data in the warm seasons, from April to September. The effects estimated from restricted data were weaker than that in models from all studied periods for respiratory diseases in Ninh Thuan in three age groups. Similar figures were observed in models of respiratory diseases in Ca Mau for ages 60+ but not for ages 6–60. In contrast, the models for CVDs in Ca Mau showed that the estimations from models in the warm season were likely stronger.

## 4. Discussion

This research assessed the relationship between heatwaves and the two heat-related health outcomes—cardiovascular and respiratory diseases. This study also fills the gap and updates the previous study on the effect of heatwaves in Vietnam. Hence, they provide scientific evidence to understand the effects of heatwaves or high temperatures on human health in Vietnam.

In this research, we found the association between respiratory admissions and heatwaves at lag 2 (ER = 8.31%, 95% CI: 0.64–16.55%) in the Ninh Thuan province. This finding reinforces existing evidence that the number of admissions for respiratory diseases increased during heatwave periods [18,21,22,23]. Indeed, a recent study has shown that heatwaves increased the number of respiratory hospitalization in Vietnam (ER = 15.5%, 95% CI: 8.00–23.0%) [18]. On the contrary, the finding in Ca Mau province did not show the association between heatwave and respiratory disease admission as several previous studies found [24,25]. Indeed, a previous study in Ca Mau also found a negative association between respiratory admissions and heatwave [26]. The difference among studies may be influenced by a data source in each study. Similar to a previous study on the effects of air pollution in Vietnam, the estimations changed depending on whether the analysis was based on provincial hospital data or national data. There were many reasons for this, including patients with severe illnesses being transferred to a high level hospital [27]. Nonetheless, further studies need to be conducted, particularly in Vietnam, to confirm this relationship.

The results illustrated a negative association between heatwave exposure and the risk of CVD hospitalization in Ca Mau. However, the association was only statistically significant for the elderly group (age above 60) (ER = −7.28, 95% CI = −13.97–−0.08). In this case, heatwave appears to be a protective factor for CVD morbidity. In the literature, the relationships between heatwaves and CVDs are mixed, and the effects of heatwaves on cardiovascular and respiratory morbidity are still unclear. A systematic review and meta-analysis pooling data from 18 studies globally has shown a statistically insignificant relationship between heatwaves and cardiovascular morbidity (*p* = 0.61) [8]. In contrast, a systematic review and meta-analysis from 64 previous studies on ambient temperature and risk of cardiovascular hospitalization by Phung et al. (2016) [28] have illustrated the risk of cardiovascular hospitalization and heatwave exposure was 2.2% (RR = 1.022; 95% CI: 1.006–1.039). In Vietnam, a study implemented in one province, and another conducted in 25 provinces also did not show a significant association between cardiovascular and respiratory diseases during and after heatwave events [28]. Unclear associations between heatwaves and CVD morbidity could be also partly explained by the immediate effect of heatwaves on mortality. Vulnerable people may be affected within several hours during a heatwave. Some of them may die before they are admitted to hospitals (out-of-hospital deaths); therefore, they will not be included in morbidity rates [29]. For example, a study by Cheng et al. (2018) [30] reported that the mortality rate suddenly increased during the first few days of heatwaves and then significantly reduced in the following days. Moreover, different subcategories of CVDs may be differently affected by changing temperatures. For instance, a study by Koken et al. (2003) [31] demonstrated that an increase in temperature was significantly associated with an increased frequency of hospital admissions for congestive heart failure and acute myocardial infarction. However, this study also indicates that the number of hospitalizations for cardiac dysrhythmias does change during heatwaves. Another possible explanation is that people, especially the elderly, may avoid going outside during heatwaves, and stay at homes with air conditioners and cooling systems [28]. One explanation that should be considered is that CVDs in general could not be a primary cause for hospital admission; therefore, studies of specific cardiovascular diseases should be investigated in further research.

Our study defines heatwave periods of at least three consecutive days when the maximum temperature exceeded the 90th percentile. In the literature, a heatwave is defined many ways including temperature indicators, intensity, and duration [16]. The authors in this study also found out that estimations vary by heatwave definitions and study design. This study observed the strongest effect at lag 4 (four days from the first day of heatwave occurrence). The models are also sensitive to choosing degrees of freedom. Moreover, to our knowledge, no previous study from Vietnam has determined heatwave threshold temperature which is essential for the health impact assessment of heatwave. Hence, we suggest Vietnam should develop a heatwave definition appropriate to local conditions to use in early warning systems and refine the models using the nonlinear distribution lag to present the health effects.

Although this study reported certain important results, some methodological limitations of this study should be acknowledged. Firstly, the study did not examine socioeconomic or air pollution factors on the association between heatwave and hospitalization data. In particular, we could not control our analysis for lifestyle (use air conditioning or not) and level of living standards (the poor or not), and comorbidities which might influence the estimation as seen in previous studies [32,33]. Second, the exposure measured in this study mainly relied on the temperature from one station in each province. These measurements might not be present for individual exposure. Moreover, the models are sensitive to choosing degrees of freedom. However, this study investigated the relationship between the change in temperature and the change in daily admission; thus, this bias could not distort the findings. Moreover, the sensitivity findings have not shown any significant difference in the effects. Third, our study mainly relies on the existing electronic database of the hospitals. The accuracy of location or patient’s address is relative since that is the patient’s reporting.

## 5. Conclusions

Associations between hospital admissions for RDs and heatwaves in Ninh Thuan were observed at lag 2, with excess risk (ER = 8.31%, 95% CI: 0.64–16.55%). However, the heatwave was negatively associated with CVDs in Ca Mau, which was determined amongst the elderly (aged above 60), ER = −7.28%, 95%CI: −13.97–−0.08%. Heatwaves can be considered a risk of hospital admission for RDs in Vietnam. Further studies are needed to assert the link between heatwaves and not only on CVDs, but other public health outcomes, particularly in terms of mortality, to identify recommendations to mitigate the negative health impacts. To mitigate health impact, Vietnam should implement urgent actions such as developing an alert and warning system or community prevention strategies.

## Figures and Tables

**Table 1:** Hospital admission for respiratory and cardiovascular diseases in Ninh Thuan and Ca Mau in total and by age group in whole studied periods and periods during heatwaves occurred.

	Entire Time ^a^						During Heatwave Occur					
	Total of Cases	Mean	SD	p^25^	p^50^	p^75^	Total of Cases	Mean	SD	p^25^	p^50^	p^75^
Ninh Thuan (1 February 2010–31 December 2017)												
Respiratory diseases (ICD10: J00–J99, exclude J60–J70)												
All	17,988	6.2	3.4	4	6	8	1369	6.3	3.3	4	6	8
6–60	5827	2.0	2.0	1	2	3	473	2.2	2.0	1	2	3
>60	6844	2.3	1.9	1	2	3	543	2.5	1.9	1	2	3
Ca Mau (31 May 2013–31 July 2018)												
Respiratory diseases (ICD10: J00–J99, exclude J60–J70)												
All	13,203	7.0	4.2	4	7	10	838	5.9	4.2	3	6	9
6–60	4480	2.4	2.1	1	2	4	287	2.0	1.9	0	2	3
>60	8722	4.6	3.0	3	4	6	551	3.9	2.9	2	4	6
Cardiovascular diseases (ICD 10: I00– I99, exclude I00–I02 and I05–I09)												
All	29,056	15.4	7.2	12	16	20	1785	12.7	7.7	9	14	18
6–60	7204	3.8	2.5	2	4	6	447	3.2	2.3	1	3	5
>60	21,851	11.6	5.7	9	12	15	1338	9.5	6.0	6	10	13

Note: ^a^ daily mean admissions were calculated across the entire study period and during heatwave days. Heatwave was defined as periods of at least three consecutive days when the maximum temperature exceeded the 90th percentile. Diseases were coded based on the ICD 10 codes in primary. Abbreviations: SD, Standard of deviation; ICD10, International Classification Diseases 10th revision.

**Table 2:** Hospital admissions for respiratory and cardiovascular diseases in Ninh Thuan and Ca Mau in total and by age group in whole studied periods and periods during heatwaves occurred.

			Average Temperature (°C)		Sun Hour (h)		Average Relative Humidity (%)		Rainfall Level (cm)	
	Number of Heatwaves	Duration	Entire Time	Heatwave	Entire Time	Heatwave	Entire Time	Heatwave	Entire Time	Heatwave
		Mean (min_max)	Mean (min_max)	Mean (min_max)	Mean (min_max)	Mean (min_max)	Mean (min_max)	Mean (min_max)	Mean (min_max)	Mean (min_max)
Ninh Thuan (1 January 2010 to 31 December 2017) ^a^										
Whole year	34	6.4 (3–17)	27.2 (21.8–31.8)	29.7 (26.5–31.8)	7.7 (0–12)	9.2 (0.1–11.7)	76.9 (50–97)	73.5 (61–88)	2.7 (0–147.7)	1.2 (0–39.2)
2010	3	7.3 (3–13)	27.4 (23–31)	29.8 (28.5–30)	8.2 (0–11.8)	9.0 (3.7–11.7)	77.6 (60–92)	75.8 (71–81)	2.8 (0–98.8)	0.3 (0–4.3)
2011	3	3.7 (3–5)	26.9 (22.6–30.4)	29.7 (29.1–30.3)	7.4 (0–11.6)	9.2 (6.5–11.4)	75.5 (57–91)	71.9 (68.0–75.0)	3.1 (0–147.7)	1.4 (0–8.2)
2012	3	7.3 (3–13)	27.3 (24–30.9)	29.0 (26.5–30.9)	7.9 (0–11.6)	8.9 (0.1–11.6)	77.1 (57–96)	74.6 (61–88)	3.2 (0–145.3)	4.3 (0–39.2)
2013	1	8	27.1 (22.5–31.2)	30.3 (29.6–31.2)	7.6 (0–11.6)	10.0 (7.4–11.6)	76.7 (58–95)	73 (70–78)	2.9 (0–65.5)	0
2014	9	5.2 (3–9)	27.1 (21.8–31.2)	29.7 (28.6–31.2)	7.9 (0–12)	8.9 (3.6–11.5)	74.9 (58–88)	71.8 (64–81)	1.4 (0–63.8)	0.9 (0–18.1)
2015	9	7 (3–17)	27.5 (21.8–31.8)	30.1 (27.8–31.8)	8.5 (0–11.5)	9.6 (7.2–11.5)	74.8 (50–92)	73 (64–80)	2.2 (0–136.6)	1.1 (0–30.8)
2016	5	8.4 (4–17)	27.5 (23.2–30.9)	29.8 (28.5–30.9)	7.5 (0–11.5)	9.0 (1–11.4)	78.7 (56–97)	74.8 (64–82)	3.5 (0–132.1)	0.7 (0–14.5)
2017	1	4	27.0 (22.4–30.5)	28.7 (28.2–29.4)	6.8 (0–11.7)	10.45 (9.9–11.2)	79.7 (55–95)	78 (77–80)	2.6 (0–76)	0
Ca Mau (31 May 2013 to 31 July 2018) ^b^										
Whole year	18	7.8 (3–37)	30.2 (28.6–32)	30.2 (28.6–32)	8.4 (2.8–11.2)	8.4 (2.8–11.2)	75.4 (68–85)	75.5 (68–85)	1.9 (0–61.2)	1.9 (0–62)
2013	0	-	27.4 (24.3–30.2)	-	4.7 (0–11.2)	-	83.5 (70–97)	-	7.3 (0–86.8)	-
2014	4	7 (3–11)	27.7 (23.2–31.1)	29.9 (28.9–31.1)	6.0 (0–11)	7.85 (2.8–11)	81.1 (66–94)	77.4 (70–85)	2.2 (0–92)	1.7 (0–19)
2015	4	1.5 (3–30)	27.9 (24.8–31.0)	29.9 (29–31)	6.5 (0–11.1)	8.4 (4.5–11.1)	80.1 (68–96)	74.8 (68–84)	6.3 (0–189.2)	2.3 (0–31.3)
2016	3	14.7 (3–37)	28.2 (24.4–32)	30.7 (28.6–32)	5.7 (0–11.2)	9.2 (5–11.2)	80.6 (64–94)	74.1 (68–83)	6.3 (0–81.5)	1.5 (0–61.2)
2017	3	5 (3–6)	27.9 (24.5–30.4)	30.2 (29.5–31.2)	5.9 (0–11)	8.3 (5.7–10.5)	79.8 (67–97)	75.6 (73–82)	4.9 (0–61.7	1.9 (0–24.4)
2018	4	3 (3–3)	27.9 (24.5–30.4)	29.8 (29.2–30.2)	5.9 (0–11)	7.1 (5–9.1)	79.8 (67–97)	77.8 (76–82)	4.9 (0–61.7)	3 (0–24.8)

^a^ Daily means of meteorological data were obtained from Phan Rang station for Ninh Thuan from 1 January 2010 to 31 December 2017. ^b^ Daily means of meteorological data were obtained from Ca Mau station for Ca Mau from 31 May 2013 to 31 July 2018. Heatwave was defined as periods of at least three consecutive days when the maximum temperature exceeded the 90th percentile.

**Table 3:** Associations between heatwaves and hospital admissions for cardiovascular and respiratory diseases in Ninh Thuan and Ca Mau, 2010–2018 at lag 2.

	Percentage Change (%)			*p*-Value
	ER (%)	95%CI *		
		Lower	Upper	
Ninh Thuan				
Respiratory diseases				
All ages	8.31	0.64	16.55	0.033 *
6–60	0.70	−11.53	14.63	0.916
60+	2.45	−8.01	14.10	0.660
Ca Mau				
Respiratory diseases				
All ages	0.53	−8.78	10.78	0.916
6–60	−3.04	−17.60	14.11	0.710
60+	2.41	−8.68	14.85	0.683
Cardiovascular diseases				
All ages	−5.11	−11.18	1.37	0.120
6–60	1.61	−9.94	14.65	0.795
60+	−7.28	−13.97	−0.08	0.048 *

While hospital data in Ninh Thuan are from 1 January 2010 to 31 December 2017, hospital data in Ca Mau are from 31 May 2013 to 31 July 2018. Exceed risk (%) = (Risk Ratio − 1) × 100%) estimated from Quasi-Poisson regression models, adjusting for long-term trends and seasonal variation, day of the week, holiday, mean humidity (natural cubic spline with four degrees of freedom). * *p* < 0.05 (Wald χ^2^ test). Abbreviations: CI: Confidence Interval.

## Data Availability

Not applicable.

## Supplementary Materials

The following supporting information can be downloaded at: https://www.mdpi.com/article/10.3390/ijerph20053908/s1, Figure S1: Map of Ninh Thuan and Ca Mau; Figure S2: The lag distribution of the associations between heatwave and hospital admissions for cardiovascular diseases in Ca Mau. Percent changes in the odds of daily hospital admissions were estimated from Quasi-Poisson regression models, adjusting for long-term trends and seasonal variation, day of the week, holiday, and mean humidity (natural cubic spline with four degrees of freedom). Hospital data in Ca Mau is from 31 May 2013 to 31 July 2018. Bar: 95% confident interval; Figure S3: Estimated percentage change of hospital admission for respiratory in Ninh Thuan at lag 2 with different degrees of freedom (knots) per year included in the cubic thin plate spline function used to capture time trends and seasonal variations. Bar: 95% confidence interval; Figure S4: Estimated percentage change of hospital admission for respiratory and cardiovascular diseases in Ca Mau at lag 2 with different degrees of freedom (knots) per year included in the cubic thin plate spline function used to capture time trends and seasonal variations. Bar: 95% confidence interval; Figure S5: the associations between heatwaves and hospital admissions for respiratory diseases and respiratory diseases in Ninh Thuan and Ca Mau for entire periods (all- downward triangle vs warm season Restrict- upward triangle). Restrict data was hospital admissions during warm seasons: from April to September. Bar: 95% confidence interval.
