# Supplementary material for: Effects of Heatwaves on Hospital Admissions for Cardiovascular and Respiratory Diseases, in Southern Vietnam, 2010–2018: Time Series Analysis
Source: Int J Environ Res Public Health. 2023 Feb 22;20(5):3908. doi: 10.3390/ijerph20053908 (PMC10001990; doi:10.3390/ijerph20053908)
Supplement: Supplementary file 1 [file ijerph-20-03908-s001.zip › ijerph-2186742-supplementary.pdf]

Effects of heatwaves on hospital admissions for cardiovascular and respiratory diseases, in Southern Vietnam, 2010-2018: Time-series analysis

Nguyen Thi Trang Nhung [1], Le Tu Hoang [1,*], Tran Thi Tuyet Hanh [2], Luu Quoc Toan [2], Nguyen Duc Thanh [4], Nguyen Xuan Truong [4], Nguyen Anh Son [4], Hoong Van Nhat [4], Nguyen Huu Quyen [5] and Ha Van Nhu [3]

[1] Faculty of Fundamental Sciences, Hanoi University of Public Health, Hanoi 10000, Vietnam
[2] Faculty of Environmental and Occupational Health, Hanoi University of Public Health, Hanoi 10000, Vietnam
[3] Faculty of Basic Medicine, Hanoi University of Public Health, Hanoi 10000, Vietnam
[4] Ministry of Health, Hanoi 10000, Vietnam
[5] Institute of Hydrology and Meteorology Science and Climate Change, Hanoi 10000, Vietnam
[*] Correspondence: lth2@huph.edu.vn; Tel.: +84-98-3-596-889

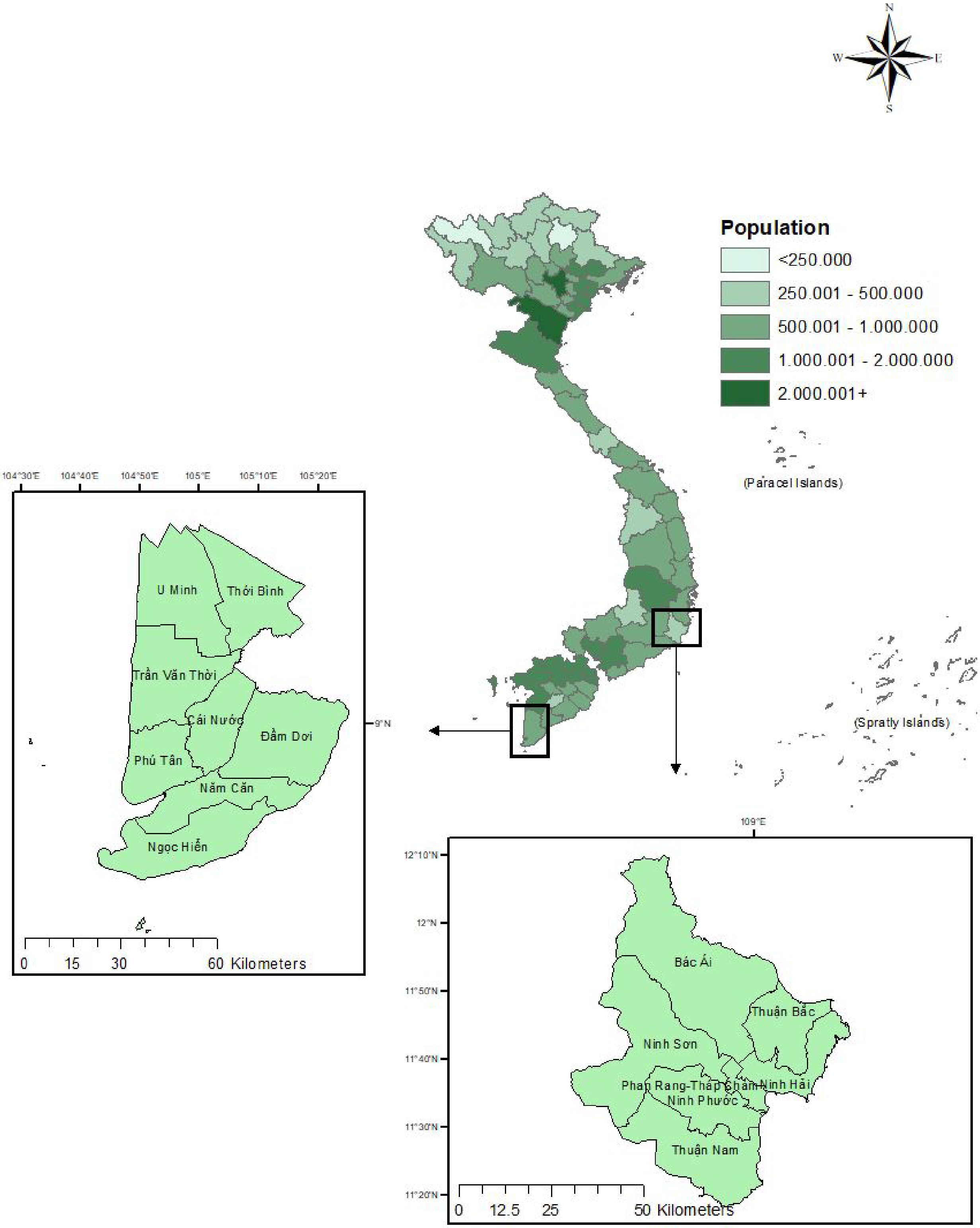

Figure S1: Map of Ninh Thuan and Ca Mau

a)

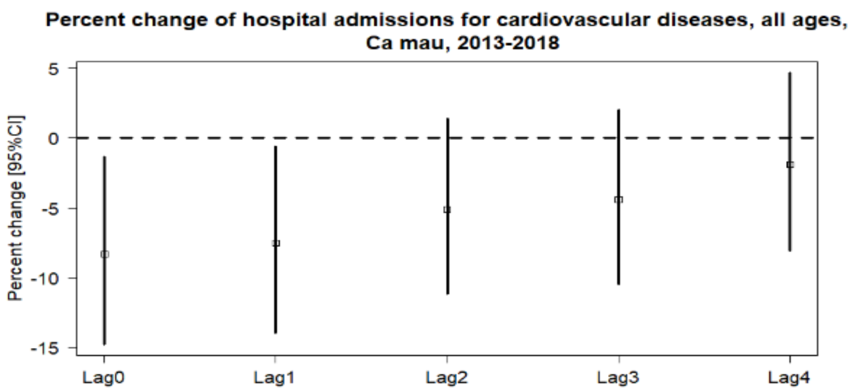

b)

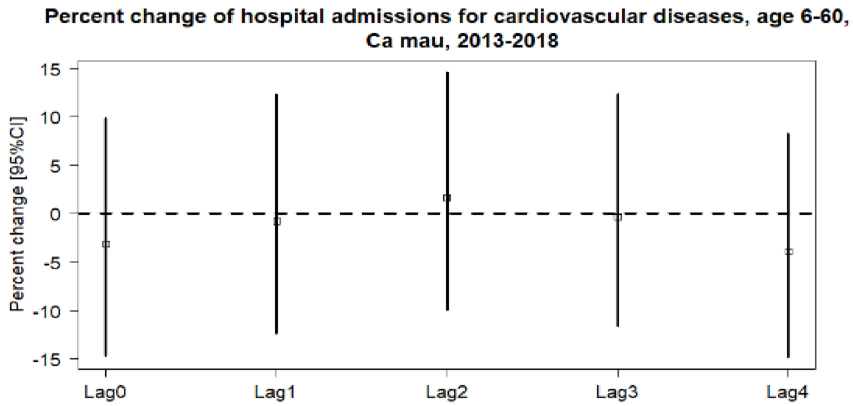

c)

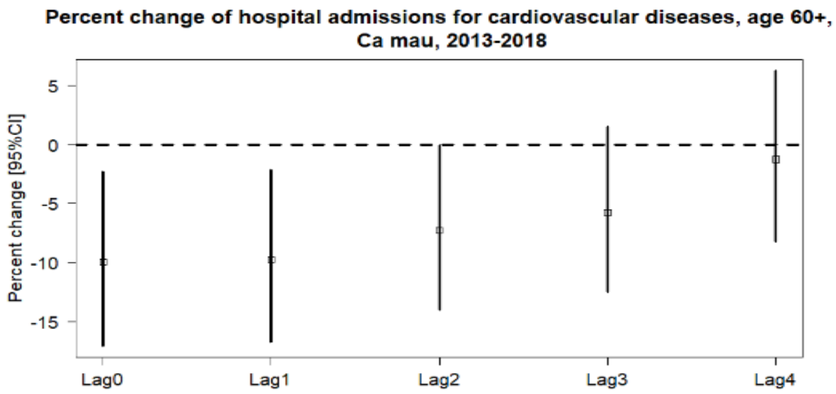

Figure S2: The lag distribution of the associations between heatwave and hospital admissions for cardiovascular diseases in Ca Mau

Percent changes in the odds of daily hospital admissions were estimated from Quasi-Poisson regression models, adjusting for long-term trends and seasonal variation, day of the week, holiday, mean humidity (natural cubic spline with four degrees of freedom).

Hospital data in Ca Mau is from 31 May 2013 to 31 July 2018. Bar: 95% confident interval

a)

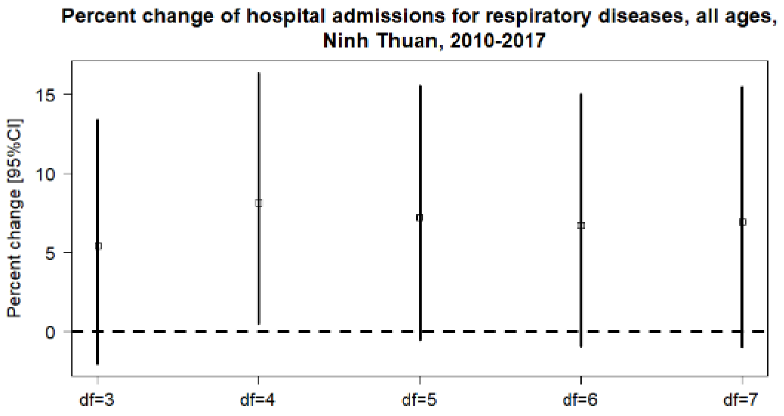

b)

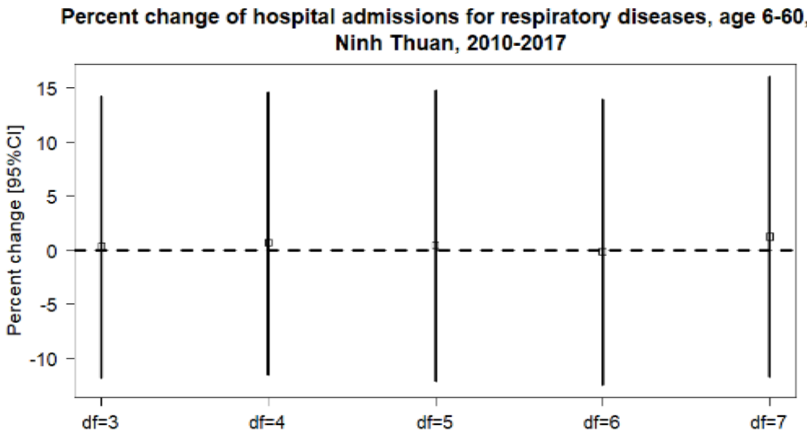

c)

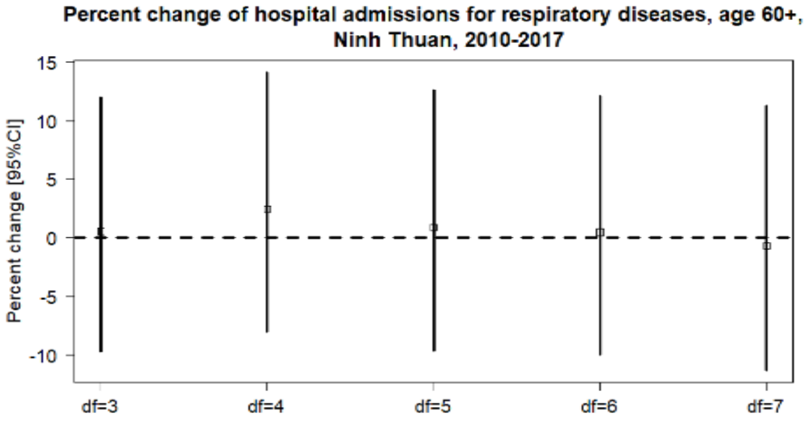

Figure S3: Estimated percentage change of hospital admission for respiratory in Ninh Thuan at lag 2 with different degrees of freedom (knots) per year included in the cubic thin plate spline function used to capture time trends and seasonal variations.

Bar: 95% confident interval

a)

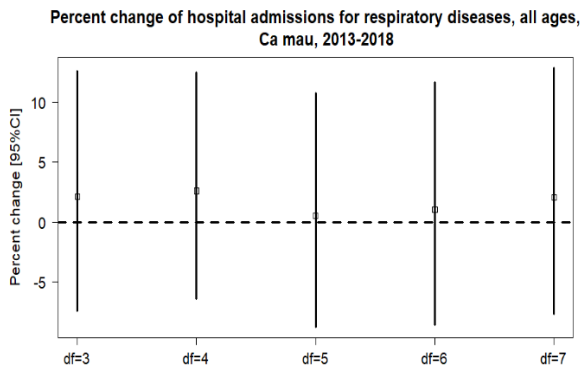

b)

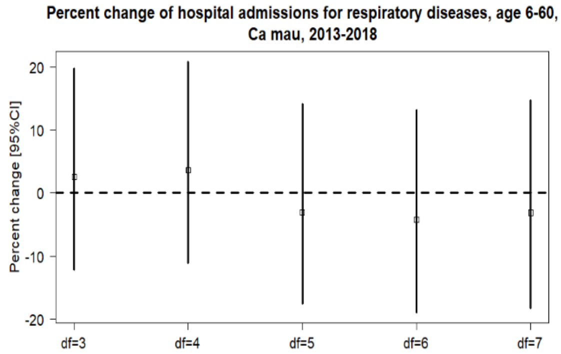

c)

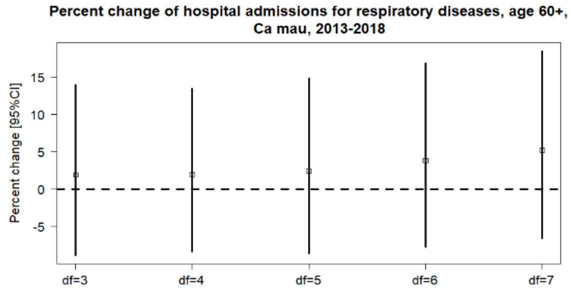

e)

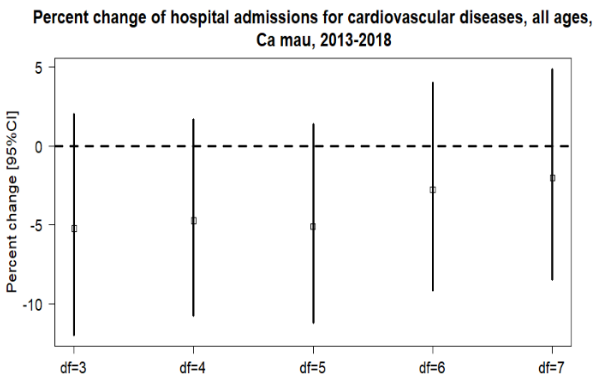

f)

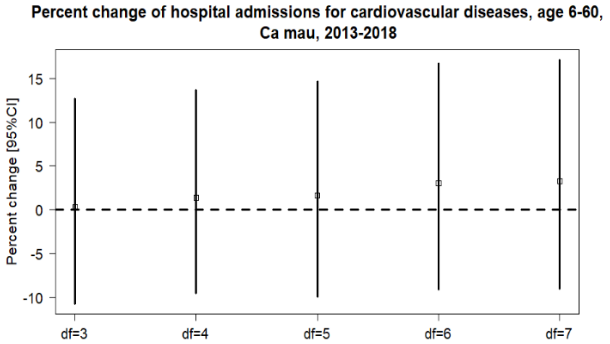

g)

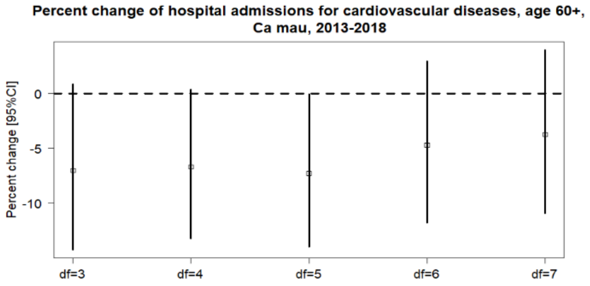

Figure S4: Estimated percentage change of hospital admission for respiratory and cardiovascular diseases in Ca Mau at lag 2 with different degrees of freedom (knots) per year included in the cubic thin plate spline function used to capture time trends and seasonal variations.

Bar: 95% confident interval

a)

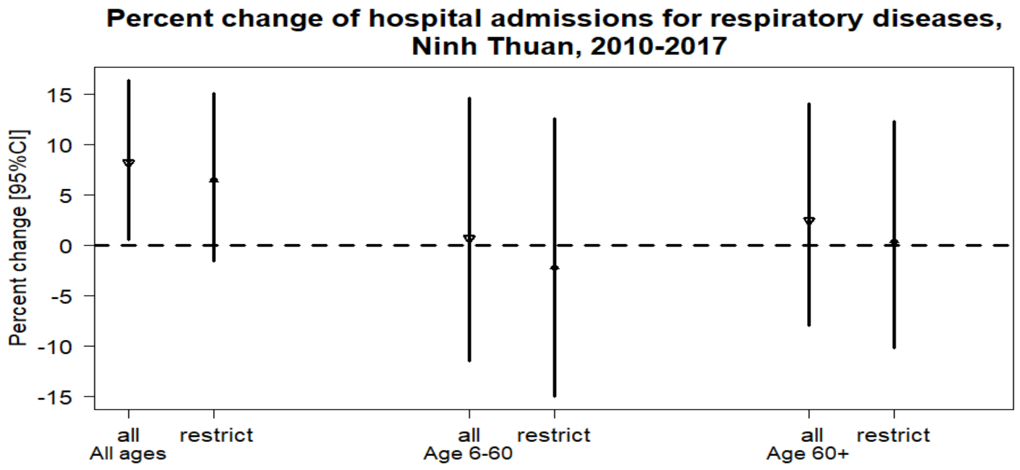

b)

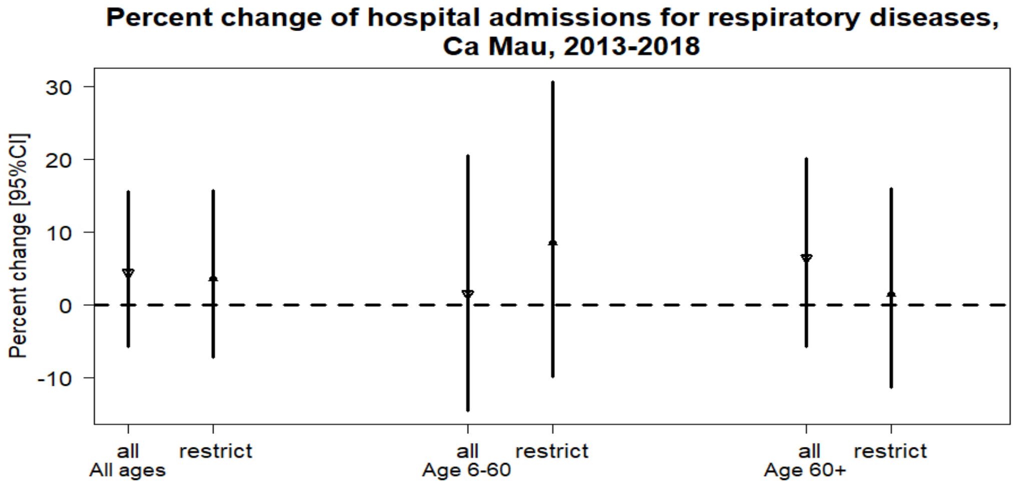

c)

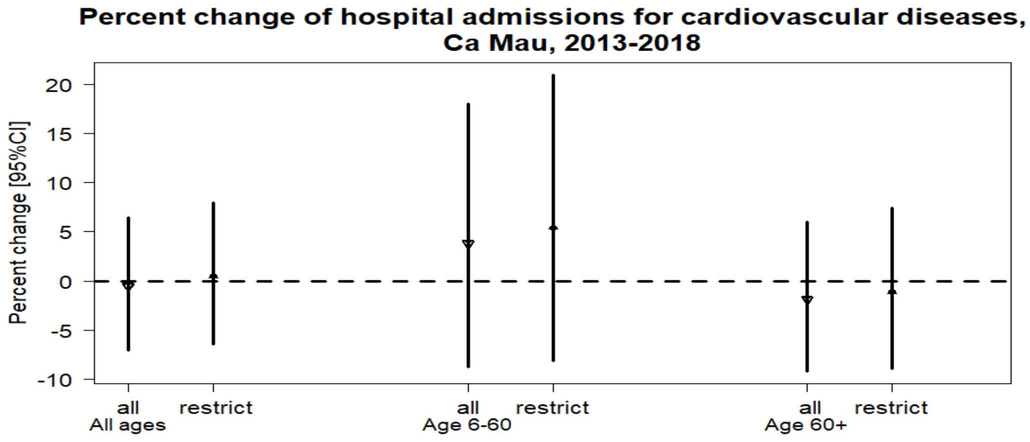

Figure S5: the associations between heatwaves and hospital admissions for respiratory diseases and respiratory diseases in Ninh Thuan and Ca Mau for entire periods (all- downward triangle vs warm season Restrict- upward triangle).

Restrict data was hospital admissions during warm seasons: from April to September
Bar: 95% confident interval
